# Supplementary material for: Acceptance and compliance with micronutrient powder (MNP) among children aged 6–23 months in northern Nigeria
Source: PLOS Glob Public Health. 2022 Oct 17;2(10):e0000961. doi: 10.1371/journal.pgph.0000961 (PMC10022258; doi:10.1371/journal.pgph.0000961)
Supplement: S1 Dataset — (ZIP) [file pgph.0000961.s008.zip › S8_Dataset/Direct Observations.zip!/Direct Observations/270615_A_GU_ID_1_DO.pdf]

1.1.1 Instrument F –Direct Observation Form

<<Instrument F Direct Observation Form>>

PI Name: PragyaMathema,Stephen Kodish
Study Title: Formative research to develop an infant and young child feeding (IYCF) intervention with micronutrient powder (MNP) for children aged 6 – 23 months
PI Version/Date: Version1.0/January 24, 2015

Date: ___27TH JUNE, 2015_________________Location: _____PUROKUYO, GUYUK L.G.A_____________

Data Collector: ___ANTIYA SANDA___________________Participant ID: _A HOUSE NEAR A BAR___

Main goals of this direct observation are to answer the following questions:

1. In this household, what would be the best way(s) to give nutritional supplements to young children? Why?

2. In this household, describe the food sharing that you observe throughout the meal. Are there any predictable patterns?

3. In this household, who is favored and how is this favoritism expressed during meal preparation and eating?

(TEAR OFF AT PERFORATED DOTTED LINE BELOW)

PARTICIPANT ID: _________

Household Composition Table (Fill out as you do data collection)

INITIALS	SEX (M/F)	AGE (estimated)	Relationship to household	Description
YW	F	25 YEARS	WIFE	A YOUNG WOMAN WEARING A GREEN WRAPPER
LB	M	19 MONTHS	SON	A LITTLE BABY
TB	M	17 YEARS	NEIGHBOUR	A TALL BLACK BOY WEARING A PURPLE SHIRT
SB	M	13 YEARS	RELATIVE	A SLIM YOUNG BOY WEARING A BLUE SHIRT
SG	F	5 YEARS	NEIGHBOUR	A SMALL GIRL WEARING A PURPLE DRESS
FB	M	35 YEARS	HUSBAND	A FAT BLACK MAN WEARING A SHIRT AND A TROUSER.

PARTICIPANT ID: __________

TIME	ACTOR INITIALS	RECIPIENT INITIALS	WHAT HAPPENED	FOOD	Reactivity
8:00	YW		SWEEPING THE HOUSE		
8:05	YW		SWEEPING THE HOUSE		
8:10	YW		SWEEPING THE HOUSE		
8:15	YW		SWEEPING THE HOUSE		
8:20	YW		SWEEPING THE HOUSE		
8:25	YW		IS GATHERING THE DIRTY PLATES TOGETHER		
8:30	YW		FETCHES WATER INTO A BIG BOWL		
8:35	YW		STARTS WASHING PLATES		
8:40	YW		IS WASHING DIRTY PLATES		
8:45	YW		FETCHES WATER IN A POT		

TIME	ACTOR INITIALS	RECIPIENT INITIALS	WHAT HAPPENED	FOOD	Reactivity
8:50	YW		STARTS MAKING FIRE		
8:55	YW		PUTS POT ON THE FIRE		
9:00	YW		BRINGS GUINEA CORN FLOUR	GUINEA CORN	
9:01	YW		STARTS SIEVING GUINEA CORN FLOUR	GUINEA CORN	
9:02	YW		CONTINUES TO SIEVE	GUINEA CORN	
9:03	YW		MIXES GUINEA CORN FLOUR WITH WATER	GUINEA CORN	
9:04	YW		POURS MIXTURE INTO THE HOT WATER ON THE FIRE	GUINEA CORN AND WATER	
9:05	YW		STARTS STIRRING THE MIXTURE ON THE FIRE	GUINEA CORN AND WATER	
9:06	YW		COVERS THE POT		
9:07	YW	LB	BRINGS HER SON FROM THE ROOM		
9:08	YW	LB	KEEPS HER CHILD ON THE MAT		
9:10	YW		OPENS THE POT ON THE FIRE TO FETCH THE KUNU	GUINEA CORN	
9:11	YW		FETCHES KUNU IN A CUP	GUINEA CORN	
9:12	YW		BRINGS A SPOON		
9:13	YW		USES THE SPOON TO MAKE THE KUNU COLD	GUINEA CORN	
9:14	YW		BRINGS DOWN THE POT ON THE FIRE		
9:15	YW		BRINGS THE CUP OF KUNU AND SPOON AND KEEPS IT NEAR HER CHILD		
9:20	YK		SITS AND ALLOWS THE KUNU TO GET COLD		
9:25	YW		GOES TO THE ROOM TO BRING THE MNP SATCHET		

TIME	ACTOR INITIALS	RECIPIENT INITIALS	WHAT HAPPENED	FOOD	Reactivity
9:30	YW	LB	BROUGHT A SATCHET OF MNP	MNP	
9:35	YW	LB	IS USING HER TEETH TO UNSEAL THE MNP SATCHET	MNP	
9:40	YW		POURS ALL THE CONTENT OF THE MNP SATCHET INTO THE CUP OF KUNU		
9:45	YW		STARTS MIXING THE MNP AND KUNU TOGETHER	MNP AND GUINEA CORN	
9:50	YW	LB	CARRYS HER CHILD ON THE LAP		
9:55	YW	LB	STARTS FEEDING HER CHILD		
10:00	YW	LB	CHECKS THE CONTENT ON THE CUP TO SEE IF THE MNP HAS BLENDED WELL		
10:00	YW	LB	IS FEEDING HER CHILD WITH THE MNP MIXED WITH KUNU	MNP AND GUINEA CORN	
10:05	YW	LB	FEEDING HER CHILD WITH MNP AND KUNU		
10:06	YW	LB	GIVES HER CHILD THE LAST SPOON		
10:07	YW		CHECKS THE CUP AND REALIZE IT IS EMPTY		
10:08	YW		HAS DRANK ALL THE KUNU MIXED WITH THE MNP		
10:09	LB		IS USING HIS TONGUE TO LICK HIS MOUTH		
10:10	YW		CARRYS EMPTY CUP TO WASH		
10:15	YW		IS WASHING THE CUP AND SPOON		
10:20	YW	LB	COMES TO CLEAN THE BODY OF HER CHILD USING A RAG		
10:25	LB		IS PLAYING ON THE MAT		
10:30	LB		IS PLAYING		
10:35	LB		IS SITTING ON THE MAT AND		

TIME	ACTOR INITIALS	RECIPIENT INITIALS	WHAT HAPPENED	FOOD	Reactivity
			PLAYING		
10:40	LB		IS PLAYING		
10:45	LB		IS PLAYING		
10:50	LB		IS PALYING		
10:55	LB		IS PLAYING		
11:00	YW	LB	CARRYS HER CHILD ON THE BACK		
11:05	LB		STARTS SLEEPING AT THE BACK OF HIS MOTHER		
11:10	YW	LB	LAYS HER CHILD ON THE MAT		
11:15	LB		IS SLEEPING		
11:20	LB		IS SLEEPING		
11:25	LB		IS SLEEPING		
11:30	LB		IS SLEEPING		
11:35	LB		IS SLEEPING		
11:40	LB		IS SLEEPING		
11:45	LB		IS SLEEPING		
11:50	LB		IS SLEEPING		
11:55	LB		IS SLEEPING		
12:00	YW		BRINGS SOME DIRTY CLOTHES TO WASH		
12:05	YW		FETCHES WATER IN A BUCKET		
12:10	YW		STARTS WASHING THE DIRTY CLOTHES USING A DETERGENT		
12:15	YW		IS WASHING SOME DIRTY CLOTHES		
12:20	YW		IS WASHING DIRTY CLOTHES		

TIME	ACTOR INITIALS	RECIPIENT INITIALS	WHAT HAPPENED	FOOD	Reactivity
12:25	YW		IS WASHING DIRTY CLOTHES		
12:30	YW		IS WASHING DIRTY CLOTHES		
12:35	YW		IS WASHING DIRTY CLOTHES		
12:40	YW		STARTS RINSING THE CLOTHES		
12:45	YW	LB	CARRYS HER CHILD FROM THE ROOM		
12:50	YW	LB	GIVES HER CHILD WATER TO DRINK	WATER	
12:55	LB		STARTS CRYING AFTER DRINKING THE WATER		
1:00	YW	LB	CARRYS HER CHILD ON THE BACK		
1:05	YW	LB	FETCHES WATER IN A BUCKET		
1:10	YW	LB	BRINGS HER CHILD DOWM AND BEGINS TO BATH HIM		
1:15	YW	LB	IS BATHING HER CHILD		
1:20	YW	LB	IS BATHING HER CHILD		
1:25	YW	LB	CLEANS HER CHILDS BODY USING A TOWEL		
1:30	YW	LB	STARTS APPLYING BODY CREAM ON THE BODY OF HER CHILD		
1:35	YW	LB	IS APPLYING CREAM ON HER CHILDS BODY		
1:40	YW	LB	IS WEARING HER CHILD CLOTHES		
1:45	YW	LB	IS WEARING HER CHILD SOME CLOTHES		
1:50	LB, SG		ARE PLAYING TOGETHER		
1:55	LB, SG		ARE PLAYING TOGETHER		
2:00	FB		COMES INTO THE HOUSE TO PICK SOMETHING AND LEAVES		

TIME	ACTOR INITIALS	RECIPIENT INITIALS	WHAT HAPPENED	FOOD	Reactivity
			IMMEDIATELY		
2:05	LB, SG		ARE PLAYING TOGETHER		
2:10	SG		FETCHES WATER IN A CUP		
2:15	SG	LB	GIVES WATER TO THE BABY TO DRINK		
2:20	LB		IS DRINKING WATER	WATER	
2:25	YW	LB	GIVES HIM KUNUN ZAKI TO DRINK THAT SHE BROUGHT FROM THE NEIGHBOURS HOUSE		
2:30	LB		IS DRINKING HIS KUNU IN A CUP	GUINEA CORN	
2:35	LB		IS DRINKING HIS KUNU	GUINEA CORN	
2:40	LB		IS DRINKING KUNU AND PLAYING	GUINEA CORN	
2:45	YW		UNTIE THE BEANS PUDDING SHE BROUGHT FROM THE NEIGHBOURS HOUSE		
2:50	YW		SERVES BEANS PUDDING IN A PLATE		
2:55	YW	LB	STATRTS HAND FEEDING HER CHILD WITH THE BEANS PUDDING	BEANS PUDDING	
3:00	YW	LB	FEEDING HER CHILD	BEANS PUDDING	
3:05	YW	LB	FEEDING HER CHILD	BEANS PUDDING	
3:10	YW	LB	GIVES HER CHILD WATER TO DRINK	WATER	
3:15	YW	LB	IS GIVING HER CHILD WATER TO DRINK	WATER	
3:20	YW	LB	IS FEEDING HER CHILD WITH BEANS PUDDING	BEANS PUDDING	
3:25	LB		STARTS HAND FEEDING HIMSELF	BEANS	
3:30	LB		TAKES A CUP OF WATER TO DRINK	WATER	
3:35	LB		LAYS ON THE MAT		
3:40	LB		STARTS SLEEPING		

TIME	ACTOR INITIALS	RECIPIENT INITIALS	WHAT HAPPENED	FOOD	Reactivity
3:45	LB		IS SLEEPING		
3:50	LB		IS SLEEPING		
3:55	LB		IS SLEEPING		
4:00	YW		FETCHES WATER IN A BUCKET TO BATH		
4:05	YW		GOES TO THE BATHROOM WITH A BUCKET		
4:10	LB		IS SLEEPING		
4:15	LB		IS SLEEPING		
4:20	LB		SLEEPING		
4:25	LB		SLEEPING		
4:30	LB		SLEEPING		
4:35	LB		SLEEPING		
4:40	LB		SLEEPING		
4:45	YW		COMES OUT OF THE BATHROOM AND ENTERS A ROOM		
4:45	SB, LB		STARTS PLAYING TOGETHER ON THE MAT		
4:50	SB, LB		PLAYING TOGETHER		
4;55	SB, LB		PLAYING TOGETHER		
5:00	YW		COMES OUT OF THE ROOM AND SITS ON A CHAIR		
5:05	YW		IS SITTING ALONE		
5:05	TB		WALKS INTO THE HOUSE		
5:10	TB, YW		SITTING AND CHATTING TOGETHER		
5:15	TB, YW		ARE CHATTING TOGETHER		
5:20	TB, YW		STILL CHATTING		
5:25	SB, LB		PLAYING TOGETHER		
5:30	SB, LB		PLAYING TOGETHER		
5:35	LB		TAKES A CUP AND DRINK WATER	WATER	
5:40	LB		IS DRINKING WATER	WATER	
5:45	LB		IS PLAYING ALONE		
5:50	YW	LB	COMES AND CARRY HER CHILD ON THE LAP		
5:55	YW	LB	IS CARRYING HER CHILD ON THE LAP		
6:00	YW	LB	IS CARRYING HER CHILD ON THE LAP		

TIME	ACTOR INITIALS	RECIPIENT INITIALS	WHAT HAPPENED	FOOD	Reactivity

TIME	ACTOR INITIALS	RECIPIENT INITIALS	WHAT HAPPENED	FOOD	Reactivity

PARTICIPANT ID: _________

Primary Questions (Complete <u>after</u> you complete data collection):

From what you have seen observing THIS HOUSEHOLD, please answer the following questions in detail:

Question	Write your <u>detailed</u> response here
1. In this household, what would be the best way(s) to give nutritional supplements such as an MNP to young children to ensure compliance?<br><br>Why?	The best way to give the MNP would be in the morning, young children are used to drinking kunu in the morning<br><br>The young child would not refuse kunu in the morning because he is introduced to kunu as a complimentary feeding since he was 6 months. He is already used to it.
2. In this household, who is favored during meal preparation or feeding?<br><br>How is this favoritism expressed?	There was no meal preparation in this household today, it was only kunu that was prepared for only the young child.<br><br>I would say that the young child is favoured in a way that his kunu was the only food prepared in this household throughout the day.

3. How could we promote the MNP supplement for this family that would attract their attention?<br><br>Who would they listen to?	It should be promoted through the mother because she stays at home all day unlike the dad who is rearly at home.<br><br>This young child would listen to his mother because she knows how to put him and make him eatall his kunu and other food brought to them from the neighbors house
4. In this household, what would be the best ways to promote the MNP but limit how much it would be shared?<br><br>Why?	The best way to promote the MNP would be through the mother but the MNP in this household could be shared.<br><br>because the mother shared food with her neighbor and the neighbor in return shared some already prepared foods with this household.

5. What other issues and concerns did you see with this family that are important for us to consider with regard to introduction of an MNP?<br><br>Why?	It appears this house hold do not have food items or food stuff in reserve to use in preparing meals.<br><br>No meal was prepared, no one could tell if they have sufficient guinea corn to use in preparing kunu for the young child in weeks to come or perhaps days to come.
6. Based on your experience observing this child today, do you believe reactivity negatively impacted your ability to see the food-related behaviors of the child?<br><br>Please explain your answer in detail.	Not in any way<br><br>There was no such thing as reactivity in this household today.
